# Effectiveness of a Structured Educational Intervention on Parents’ Knowledge, Perception, and Acceptance Towards Human Papillomavirus Vaccine

**DOI:** 10.1002/puh2.70076

**Published:** 2025-07-04

**Authors:** Radha Devi Dhakal, Sudipa Poudel, Pushpa Sigdel, Sarmila Regmi

**Affiliations:** ^1^ Department of Nursing Shree Medical and Technical College Bharatpur Nepal; ^2^ Narayani Vidya Mandir Bharatpur Nepal; ^3^ Shree Medical and Technical College Bharatpur Nepal

**Keywords:** human papillomavirus (HPV) vaccine, knowledge and perception, parents, acceptance and Nepal

## Abstract

**Background:**

Cervical cancer is one of the common cancer among women worldwide, with high mortality and morbidity rates. The human papillomavirus (HPV) vaccine is one of several preventative options for cervical cancer. The HPV vaccine is just introduced in Nepal and is still not included in the national immunization schedule. Awareness courses for parents may be beneficial in overcoming their hesitancy. This study aims to assess the effectiveness of a structured educational intervention on parents’ knowledge, perception, and acceptance towards HPV vaccine.

**Methods:**

An interventional one‐group pretest and posttest study design was used among selected parents whose daughters are currently studying in classes 5–7 (Ages 9–12 years) in the schools of Bharatpur metropolitan. Multistage sampling technique was used to select sample population. Pre‐ and post‐intervention data were collected between January 1, 2023 to February 30, 2023‐through a structured self‐administered questionnaire in the structured‐educational intervention program. Collected data were analyzed by using Statistical Package of Social science version 22.

**Results:**

There was a significant difference between pre‐and post‐educational intervention parents’ knowledge and perception of HPV infection. The result showed a significant increase in knowledge scores before (mean 7.02, SD 3.11) to after (mean 27.81, SD 4.20), *t*(−75.97), *p* ≤ 0.001. Similarly, a significant increase in perception scores before (mean 27.81, SD 4.20) to after (mean 41.50, SD 2.91), *t*(−49.95), *p* ≤ 0.001. Acceptance of HPV vaccine was also increased in posttest (40.3%–87.9%).

**Conclusion:**

After the structured‐educational intervention, there were significant improvements in parents’ knowledge, and perception, with increasing acceptance of the HPV vaccine. A structured educational intervention designed for parents may have important implications for improving vaccine acceptability. Awareness program about regarding HPV infection and the HPV vaccine would be beneficial to raise the acceptance of vaccine.

## 1 Background of the Study

Cervical cancer is a type of cancer that occurs in the cells of the cervix, the lower part of the uterus that connects to the vagina [1]. Cervical cancer is one of the common cancer among women worldwide, with high mortality and morbidity rates [2, 3]. Worldwide, cervical cancer is the fourth most frequent cancer in women with an estimated 604,000 new cases and 342,000 deaths in 2020 [2]. About 90% of cervical cancer cases occur in low‐ and middle‐income countries. Age‐standardized incidence rates of cervical cancer are highest in Asia that is, 58.2%. In Nepal, cervical cancer is the leading cause of female cancer with 2244 new cervical cancer cases with 16.4% age‐standardized incidence rates [4, 5]. Human papillomavirus (HPV) types 16 and 18 are a common cause of cervical cancer and there is growing evidence of HPV being a relevant factor in other anogenital cancers (anus, vulva, vagina, and penis) and head and neck cancers [4, 5, 6].

Vaccines are one of the most reliable and cost‐effective public health interventions ever implemented, saving millions of lives each year [7, 8]. The HPV vaccine is highly effective for the prevention of HPV serotypes 16 and 18, which cause 70% of cervical cancer [4]. HPV vaccines have been demonstrated to be remarkably effective and safe mainly to prevent the development of high‐grade cervical cancer [9]. Positive trends in decreasing high‐risk HPV infections and pre‐cancerous HPV‐related lesions in vaccinated individuals have been observed in recent years [10].

Cervical Cancer Elimination Initiative was launched by WHO to address several challenges, including the inequity in vaccine access. Broad distribution and acceptance of vaccines are required to achieve the goal of having 90% of girls vaccinated by the age of 15 by 2030 [11]. Globally, the uptake of the life‐saving vaccine has been slow, and coverage in low‐ and middle‐income countries is much lower. In 2020 global coverage with two doses of HPV was only 13% [11].

Children aged 9–14 years’ age group need parental consent for the vaccination, so the acceptance of vaccine is depending on parental approval [12]. Educating parents was demonstrated to be an effective method for improving vaccine acceptability by increasing their knowledge and positive perceptions towards vaccination [13]. Assessing a short education seminar designed for low‐educational caregivers showed a remarkable improvement in vaccination knowledge [14]. Despite sound scientific data supporting the vaccine's efficacy, most parents hesitate to get their children vaccinated [15]. There is a paucity of data regarding parental acceptance of HPV vaccination in developing countries [16]. The HPV vaccine is just introduced in Nepal and is still not included in the national immunization schedule. So, the study aims to identify the effectiveness of a structured educational intervention on parents’ knowledge, perception, and acceptability towards the HPV vaccine.

## 2 Research Methods

### 2.1 Research Design

An interventional one‐group pretest posttest study design was used in the selected schools of Bharatpur metropolitan.

#### 2.1.1 Research Setting and Population

Bharatpur, the district headquarter of the Chitwan District and a distinct Metropolitan Authority, has a population of 199,867. Bharatpur is located in central‐southern Nepal, about 146 km from Kathmandu [17]. It is also renowned as Nepal's educational hub city. There are 109 government schools and 95 private schools that offer education from grades 4–12 [18].

#### 2.1.2 Population

Mother parent's daughters are currently studying in classes 5, 6, and 7 (ages 9–12 years). Inclusion criteria were parents of those daughters who have not taken any dose of the HPV vaccine and voluntary agreement to participate in the present study. Parents with speaking, hearing, and visual impairments and who are unable to read and write were excluded.

#### 2.1.3 Sample Size

The sample size was calculated on the basis of 27% [19], prevalence, za = 1.96, *e* = 0.05% *q* = (1 − *p*) = 1 − 0.27 = 0.73. By using the formula(*n*) = zα^2^pq ÷ *e* ^2^, the calculated sample size was 302.8, Adding a 10% non‐response rate the required sample size was 333.

#### 2.1.4 Sampling Technique

A multistage sampling technique was used. Initially, the 29 wards of Bharatpur Metropolitan City were grouped into three separate categories on the basis of number of schools and geographical location. Out of three separate categories, two wards were randomly chosen from each, resulting in a total of six wards selected. We gathered a list of schools that have school nurses from the provided list of wards. Afterward, randomly schools were selected. In the selected schools a census was conducted to identify parents who fulfilled the inclusion criteria and a list of population frames was created. Finally, Parents of girl students in grades 5, 6, and 7 (9–12‐year old) were selected using a simple random sampling technique until the sample size meet.

#### 2.1.5 Data Collection Tool

The researchers developed a structured self‐administered questionnaire after an extensive literature review [20, 21, 22] and consultation with subject experts. The research instruments consisted of three parts: Part I: Questions related to socio‐demographic information included age, ethnicity, religion, education, occupation, history of cervical cancer and sources of information.

Part II: Knowledge regarding sexually transmitted infection (STI), cervical cancer, HPV infection, and HPV vaccine, which had 24 questions. These questions were answered on a yes or no basis. A correct answer was worth one point, whereas an incorrect/unknown answer received zero points. The total knowledge score varied from 0 to 24, Pre intervention score ranged (min 3–max 16) with 7.02 mean score and post‐knowledge (min 15–max 22) with 19.91 as a mean score, where higher score indicating better knowledge cutoff score was mean value and Part III includes parental perception towards HPV vaccination that were measured by a five‐point Likert scale. Positive statements were scored from 5 = strongly agree to 1 = strongly disagree, whereas negative statements were reverse score. It contains total 8 items, the total score was 40, pre‐intervention score was (min 20–max 42) 27.81 as mean score, and post intervention perception (min 34–max 49) 41.5 as mean score, cutoff score was mean value, above that indicates a favorable attitude.

The content validity of the tool was established by thoroughly reviewing the literature and consultation with subject experts. The tool was developed in the English language, translated from English to Nepali, and back‐translated to English. For comprehensibility and simplicity of language, suggestions were taken by a language expert. The research instrument was pretested among 10% of mothers from different settings, and necessary modifications were made.

#### 2.1.6 Ethical Considerations

Participants have explained the goal and objectives of the study in clear and understandable terms. Before data collection, confidentiality was assured by not disclosing the information except for the study purpose. Participants were allowed to withdraw from the study if they wanted.

#### 2.1.7 Data Collection Procedure

The data were collected through a structured self‐administered questionnaire. A study information statement was sent to the parents through students, and they were invited to participate in the study with the help of a school health nurse. The objectives of the study were clearly stated.

#### 2.1.8 Intervention

A PowerPoint (PPT) presentation session provided by a trained school health nurse. The educational content and presentation materials was adopted and modified as relevant to the target population according to context after pretest. Both pre and post intervention had the same content that covered the following: sexually transmitted diseases, cervical cancer, HPV infection, and HPV vaccination and its indication, as well as the benefits and safety of HPV vaccination, including common misconceptions. Each education intervention group consisted of 30–40 parents. After a short introductory session, socio‐demographic information and baseline data were gathered. After that, a pretest questionnaire was distributed to the participants. The participants were given approximately 10–15 min to complete the questionnaire and then it was collected. The intervention consists of a PowerPoint (PPT) presentation provided by trained school health nurses in a simple understandable language; followed by an interactive discussion session. The session lasted around an hour. The post‐intervention data were collected immediately after the educational intervention.

#### 2.1.9 Data Analysis Procedure

Collected data were checked, reviewed, and organized for accuracy and completeness daily by the researcher. Three incomplete questionnaires were omitted, and only 330 completed questionnaires were used for further analysis. The responses in the completed questionnaires were coded and entered in Excel 16 and exported to SPSS version 22 for analysis. It was cleaned and edited (checking for missing values and outliers). Data analysis was done using descriptive (frequency, mean, and standard deviation) and inferential statistical methods (paired sample *t*‐test). After that, the conclusion of the study was drawn and presented in the tables and figures.

## 3 Results

Table 1 shows that regarding age out of 330 respondent's majority, 43.3% of respondents belong to the age group of (31–35) years. The mean value and SD of age were mean = 32.01, SD ± 5.84, min 23 and max 63. Concerning religion, the majority 83.3% of respondents followed Hinduism, and 40.3% were Janajati. At the same time, concerning education, majority 35.5% had taken basic education, and 57.3% had monthly family income, below Rs. 20,000 incomes. The majority 47.9% of the respondents had two children. Most importantly, a family history of cervical cancer was not found in the majority of respondents (94.5%). Furthermore, the source of information on HPV infection and HPV vaccine for a considerable number of respondents, that is, 47.2%, turned out to be health workers, and 35.5% mass/media.

**TABLE 1:** Respondents' socio‐demographic information *n* = 330.

Variables	Frequency	Percentage
**Age group (in years)**		
20–25	18	5.5
26–30	70	21.2
31–35	143	43.3
36–40	76	23.0
41–45	13	3.9
>45	10	3.0
Mean = 32.01, SD = 5.82, Min 23 and Max 63		
**Education level**		
General literate (can read and write)	104	31.5
Basic education (1–8)	117	35.5
Secondary education (9–12)	62	18.8
Bachelor's education and above	47	14.2
**Religion**		
Hinduism	275	83.3
Buddhism	30	9.1
Christianity	22	6.7
Islam	3	0.9
**Ethnicity**		
Dalit	73	22.1
Janajati	133	40.3
Madhesi	4	1.2
Muslim	3	0.9
Brahmin/Chhetri	106	32.1
Others	11	3.3
**Occupation**		
Farmer	153	46.4
Homemaker	34	10.3
Business	47	14.2
Service holder	96	29.1
**Monthly family income NRs**		
Up to 20,000	189	57.3
21,000–40,000	109	33
41,000–60,000	23	7
>60,000	9	2.7
**Number of children**		
One child	22	6.7
Two children	158	47.9
Three children	123	37.3
Four children	22	6.7
More than four	5	1.5
**Family history of cervical cancer**		
Yes	18	5.5
No	312	94.5
**Source of information**		
Health worker	143	47.2
Internet	107	35.3
TV	87	28.7
Relatives’ friends	84	27.7
Radio, FM	80	26.4

Table 2 shows overall increment in knowledge after educational intervention. Before the educational intervention, only a few of the parents knew about the meaning of STIs 30.6% and cervical cancer 26.7% in the posttest increased by 95.2% and 85.5%. Regarding types of STIs most of the respondents 24.4% correctly responded that HPV in the posttest increased by 91.5%. However, only 26.7% of respondents knew the HPV is the main cause of cervical cancer that has increased by 96.7% in the posttest. Likewise, on the question about other disease conditions caused by HPV, the majority of respondents gave a response on oral cancer at 40.9% and increased in the posttest by 95.2%. Concerning the HPV transmission majority 52.1% responded response correctly which increased by 97.6% in the posttest. When asked about preventive measures for HPV infection majority, 57.9% responded response on avoiding multiple sex partners and this increased by 98.8% in the posttest. On the statement, HPV vaccine is used to prevent cervical cancer 32.4% gave correct response and increased in posttest by 97%. Regarding the appropriate time for the HPV vaccine, 39.4% gave correct response after the posttest increased by 78.5%; likewise response on the appropriate age for HPV vaccine between 9 and 26 years was 27.3%, and after the posttest increased by 85.2%. Similarly, while asking about the recommended dose for the HPV vaccine, 35.5% gave the correct response, and after the posttest increased by 96.1%. On the availability of the HPV vaccine, 67.6% said it is not free, and after the posttest increased by 93.9%. Regarding acceptance of HPV vaccine pretest acceptance 40.3% and posttest acceptance is increased in 87.9%.

**TABLE 2:** Respondents’ knowledge regarding human papillomavirus (HPV) infection and HPV vaccine *n* = 330.

Knowledge items	Correct answer	Pretest	Posttest
Meaning of sexually transmitted infections	Infections that are passed from one person to another through sexual contact	101 (30.6)	314 (95.2)
Sexually transmitted infections (STIs) are	Human papillomavirus (HPV) infection	81 (24.4)	302 (91.5)
	Human immunodeficiency virus (HIV) infection	66(20)	310(93.9)
	Syphilis infection	20(6.1)	302(91.2)
	Gonorrhea infection	19(5.8)	236(71.5)
Cause of STIs	Having unsafe sex with STIs Person	139(42.1)	250(75.8)
Meaning of cervical cancer	Cancer that occurs in the cell of the cervix	162(49.1)	283(85.5))
Cause of cervical cancer	Human papillomavirus (HPV)	88(26.7)	319(96.7)
Other disease conditions caused by Human papillomavirus	Anal cancer	94(28.5)	304(92.1)
	Genital warts	39(11.8)	313(94.8)
	Penile cancer	43(13)	268(81.2)
	Vaginal cancer	134(40.6)	305(92.4)
	Oral cancer	135(40.9)	314(95.2)
HPV transmitted	Through unsafe sexual contact	172(52.1)	322(97.6)
Preventive measures of HPV infection	Avoid multiple sex partner	191(57.9)	326(98.8)
	Safe sex practice	160(48.5)	318(96.4)
	HPV vaccination	104(31.5)	305(92.4)
HPV vaccine	Vaccine which is used to prevent cervical cancer	107(32.4)	320(97)
Appropriate time for HPV vaccine	Before becoming sexually active	130(39.4)	259(78.5)
Appropriate age for HPV vaccine	Between 9 and 26 years	90(27.3)	281(85.2)
Recommended dose for HPV vaccine	Two dose	117(35.5)	317(96.1)
HPV vaccine is free of cost	No	223(67.6)	310(93.9)
Acceptance of vaccine	Yes	197(40.3)	290(87.9)

Table 3 summarizes the overall respondents’ perceptions regarding HPV infection and HPV vaccine after educational intervention. In positive statement score “agree” and “strongly agree” score were combined and in negative statement “disagree” and “strongly disagree” were summed. Before the educational intervention, only a few of the parents, 17.8%, agreed with the statement “HPV infection is a sexually transmitted infection that affects the genital area,” after educational intervention increased by 79.7%. Regarding the statement, “I trust the information I receive about HPV vaccines,” 26.6% of respondents agreed. Overall, 89.4% increased after educational intervention. Concerning the negative statements “I am worried that vaccines cause long‐term negative effects to my daughter,” 14% were disagree and 89.4% increased after educational intervention. Regarding the negative statement, “I do not believe vaccines are safe, only 29.1% gave correct responses by disagreeing with the statements and 93.1% increased after an educational intervention.” On the statement “HPV vaccine is most effective if it is given before HPV exposure” 27.9% agreed 88.2% increased after educational intervention. Regarding the statement that the “HPV vaccine is most effective if it is given before HPV exposure,” 37.6% agreed and 88.1% increased after an educational intervention. Regarding the statement, “HPV vaccine does not give protection from cervical cancer” only 37.6% gave a correct response by disagreeing the statement and 88.1% increased after an educational intervention. Regarding the statement, “I believe that HPV vaccination is useful for preventing cervical cancer,” 30.3% agreed and 86.1% increased after educational intervention. Regarding the statement, “I think school girls are not at risk of HPV infection,” 19.1% gave a correct response by disagreeing with the statements 92.5%.

**TABLE 3:** Respondents’ perception regarding human papillomavirus (HPV) infection and HPV vaccine *n* = 330.

Perception statement	Pretest	Posttest
	SA/A	SA/A
HPV infection is a sexually transmitted infection that affects the genital area	59(17.8)	263(79.7)
I trust the information I receive about HPV vaccines	88(26.6)	311(94.4)
I am worried that vaccines cause long‐term negative effects to my daughter	79(14.0)	295(89.4)
I do not believe vaccines are safe	96(29.1)	307((93.1)
HPV vaccine is most effective if it is given before HPV exposure	92(27.9)	291(88.2)
HPV vaccine does not give protection from cervical cancer	124(37.6)	281(88.1)
I believe that HPV vaccination is useful for preventing cervical cancer	100(30.3)	284(86.1)
I think school girls are not at risk of HPV infection	63(19.1)	305(92.5)

Abbreviations: A, agree, SA, strongly agree.

**FIGURE 1:**
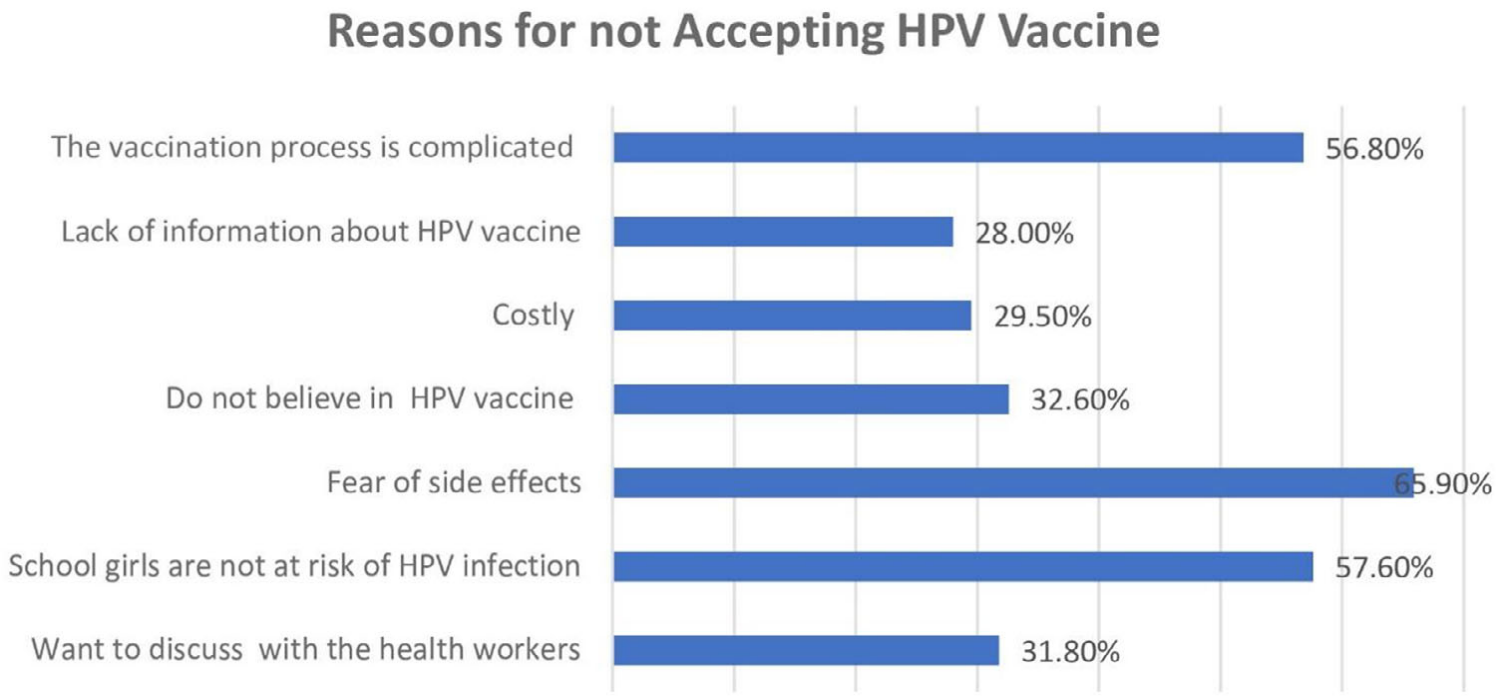
Reasons for not accepting HPV vaccine. Shows the reasons for not accepting the HPV vaccine, where most of the parents, 65.90% rejected the vaccine due to fear of side effects. Other reasons for the refusal were school girls not at risk of HPV infection 57.60%, the vaccine process being complicated 56.80%, not believing in the HPV vaccine 32.60%, wanting to discuss with health workers 31.80%, costly 29.50% and lack of information 28%. HPV, human papillomavirus.

In Table 4, a paired sample *t*‐test was conducted to evaluate the impact of pretest and post scores of knowledge and perception on HPV infection and HPV vaccination. The result showed a significant increase in the scores before (mean 7.02, SD 3.11) to after (mean 27.81, SD 4.20), *t*(−75.97), *p* ≤ 0.001(two‐tailed). The mean increase in knowledge test scores was −12.89. Similarly, a significant increase in the perception scores before (mean 27.81, SD 4.20) to after (mean 41.50, SD 2.91), *t*(−49.95), *p* ≤ 0.001(two‐tailed). The mean increase in perception test scores was −13.69 (Figure 1).

**TABLE 4:** Difference in knowledge and perception before and after educational intervention.

Variables	Mean SD (pretest)	Mean SD (posttest)	Difference	Standard deviation	*t* value	*p* value
Knowledge	7.02(3.11)	19.91(1.81)	−12.89	3.08	−75.97	<0.001
Perception	27.81(4.20)	41.50(2.91)	−13.69	4.98	−49.95	<0.001

## 4 Discussion

This study revealed that short educational intervention raised the overall knowledge perception and vaccine acceptability. Previous study showed pre‐intervention, levels of awareness and knowledge of HPV, cervical cancer, and preventive measures were found to be low [22, 23, 24, 25, 26, 27, 28, 29]. Similarly, in this study, majority parents have low knowledge on HPV vaccine that were marked increase after educational intervention. Educational interventions are crucial to improve the parents’ knowledge about HPV infection and vaccination [30]. Educating parents was demonstrated to be an effective method to improve vaccine acceptability by increasing their knowledge and positive perceptions towards vaccination [31, 32]. Short interactive education program with PowerPoint slides and recorded videos to introduce the HPV vaccine showed a remarkable improvement in vaccination knowledge [33, 34]. Studies during the COVID‐19 pandemic concluded that educational health programs play a vital role in promoting the knowledge attitudes and preventive practices [35]. In our study, post intervention knowledge and perception were raised. The possible explanation might be education makes mother more concerned for their child's health and more aware on preventive measure.

Like knowledge pre intervention level of attitude about HPV and HPV vaccination was found to be low in this study. Most of the parent have unfavorable perception like HPV infection is an STI that affects the genital area only, long‐term effects and vaccine safety and school girls are not at risk of HPV infection. In contradictory, few study suggested that the parents were concerned that their child could contract the infection and believed that the vaccination was useful for the prevention [9, 30]. Parents’ attitudes and perception were influenced by knowledge and education this indicates that parents need to be educated [2, 15, 19, 30, 36, 37].

In our study, most reported source of information about HPV being healthcare providers and the next most popular was the internet and social media. Parents received information about HPV vaccination from a myriad of sources but overwhelmingly through health‐care providers [17, 38]. Studies suggested that parents who had received information from a physician were more likely to consider HPV vaccination useful for the prevention of HPV diseases [3, 16]. Healthcare workers are considered influential and a key determinant of HPV vaccine acceptance and compliance and social media is also a powerful source of information maximum of the participants received their information from social media [28]. World Health Organization (WHO) understands that the barriers and facilitators to HPV adolescent vaccine programs in LMICs may help increasing HPV vaccine acceptance, uptake, and coverage [34]. WHO is now encouraging for all countries, particularly LMICs, to introduce HPV vaccines into their routine immunization programs [2]. Recently Nepal has also started HPV vaccination program but still not included in national immunization schedule. Like previous studies [1, 20] findings in this study post‐intervention, willingness to vaccinate their children increased. Study suggested that better knowledge and positive perceptions of the HPV vaccination were significantly associated with higher acceptability [21, 39, 40]. Post‐intervention, respondents were twice as likely to report willingness to vaccinate their children [20]. The study showed that significant proportion of parents willing to protect their daughters from being infected with HPV in the future [41]. The most common reasons for parents’ refusal to vaccinate their children are fear of adverse effects and the belief that schoolgirls are not at danger of HPV infection, which is corroborated by other studies [20, 41]. Almost half of respondents (49% and 41.8%) were concerned that the HPV vaccine will not prevent the disease [23, 42]. In this study, parents’ willingness to vaccinate was rather high, and cost did not appear to be a significant obstacle. Information on vaccine safety and efficacy is vital and parents need information about HPV and the HPV vaccine [23, 42]

The study was confined to the parents of school girl studying in class 5–7 in Bharatpur metropolitan city only, so findings cannot be generalizable in all places. One‐group pretest–posttest design study without a control group did not allow to confirmation of any causal relations between the intervention and outcomes. Further true experiment study designs are needed to evaluate the long‐term and actual effectiveness of improving parents’ knowledge and perceptions about the HPV infection and HPV vaccine as well as the vaccine acceptability. Confounding bias may occur due to unmeasured variables.

## 5 Conclusion

A structured‐educational intervention may improve parental knowledge, perception and acceptance of HPV infection and HPV vaccine. There was a significant difference between pre‐ and post educational intervention on parents’ knowledge and perception of HPV infection and HPV vaccine with increasing acceptability of the HPV vaccine. HPV vaccination program helps to prevent cervical cancer and deaths and likely reduce overall health care costs. Therefore, proper dissemination of information regarding HPV infection and the HPV vaccine, and the removal of misperceptions about the HPV vaccine is necessary to raise the acceptance.

## Author Contributions

**Radha Devi Dhakal**: conceptualization, methodology, software, data curation, formal analysis, visualization, funding acquisition, investigation, writing – original draft, writing – review and editing, resources. **Sudipa Poudel**: investigation, writing – review and editing, data curation, funding acquisition, resources. **Pushpa Sigdel**: investigation, writing – review and editing, data curation. **Sarmila Regmi**: funding acquisition, writing – review and editing, formal analysis, supervision, resources, validation, visualization, project administration, software, methodology.

## Ethics Statement

Ethical approval was obtained from SMTC‐IRC‐202200626‐28; formal permission was obtained from the concerned authorities for data collection.

## Consent

Written informed consent was obtained prior to data collection.

## Conflicts of Interest

The authors declare no conflicts of interest.

## Data Availability

The data that support the findings of this study are availability from the corresponding author on reasonable request because the scope of the data and consent obtained from study participants restricts our ability to share the data on ethical and legal rules.
